# Neuroprotection by Endoplasmic Reticulum Stress-Induced HRD1 and Chaperones: Possible Therapeutic Targets for Alzheimer’s and Parkinson’s Disease

**DOI:** 10.3390/medsci4030014

**Published:** 2016-08-18

**Authors:** Jun Nomura, Toru Hosoi, Masayuki Kaneko, Koichiro Ozawa, Akinori Nishi, Yasuyuki Nomura

**Affiliations:** 1 RIKEN Brain Research Institute, Saitama 351-0198, Japan; Jun.nomura@riken.jp; 2 Department of Pharmacotherapy, Graduate School of Biomedical and Health Sciences, Hiroshima University, Hiroshima 734-8553, Japan; toruh@hiroshima-u.ac.jp (T.H.); ozawak@hiroshima-u.ac.jp (K.O.); 3 Department of Biochemistry, Graduate School of Biomedical and Health Sciences, Hiroshima University, Hiroshima 734-8553, Japan; mkaneko@hiroshima-u.ac.jp; 4 Department of Pharmacology, Kurume University School of Medicine, Kurume 830-0011, Japan; nishia@med.kurume-u.ac.jp

**Keywords:** endoplasmic reticulum, unfolded protein responses, HRD1, molecular chaperone, degradation/refolding of misfolded proteins, Alzheimer’s disease, Parkinson’s disease, therapeutic development

## Abstract

Alzheimer’s disease (AD) and Parkinson’s disease (PD) are neurodegenerative disorders with a severe medical and social impact. Further insights from clinical and scientific studies are essential to develop effective therapies. Various stresses on the endoplasmic reticulum (ER) cause unfolded/misfolded proteins to aggregate, initiating unfolded protein responses (UPR), one of which is the induction of neuronal cell death. Some of the pathogenic factors for AD and PD are associated with UPR. ER molecules such as ubiquitin ligases (E3s) and chaperones are also produced during UPR to degrade and refold aberrant proteins that accumulate in the ER. In this review, we examine the role of HMG-CoA reductase degradation protein 1 (HRD1) and the chaperone protein-disulfide isomerase (PDI), which are both produced in the ER in response to stress. We discuss the importance of HRD1 in degrading amyloid precursor protein (APP) and Parkin-associated endothelin receptor-like receptor (Pael-R) to protect against neuronal death. PDI and the chemical chaperone 4-phenyl-butyrate also exert neuroprotective effects. We discuss the pathophysiological roles of ER stress, UPR, and the induction and neuroprotective effects of HRD1 and PDI, which may represent significant targets for novel AD and PD therapies.

## 1. Introduction

The prevalence of dementia is estimated to be approximately 25,000,000 people worldwide and is increasing exponentially as the number of senescent individuals increase. More than half of dementia patients have Alzheimer’s disease (AD). Another serious neurodegenerative disease is Parkinson’s disease (PD). Medical and social therapies are required for both diseases. Elucidating the pathogenic mechanisms underlying AD and PD is essential for treatment and will pave the way for the development of novel therapeutics.

Several genes are known to be involved in familial AD and PD, while epigenetic contributions to the pathogenesis have not yet been elucidated. A number of environmental, biological, chemical, and physical factors can cause pathological states. Metabolic abnormalities associated with diabetes mellitus type II and atherosclerosis/hyperlipidemia have been implicated in AD and PD. Hypertension has also been identified as a risk factor. Although the pathogenesis of neurodegeneration involves a complex combination of genetic and epigenetic factors, a common mechanism may be neuronal death in the brain. Therefore, the cerebral vascular systems in the supply of glucose and oxygen and the astroglial and microglial cells in the maintenance of neurons, have important roles in preventing neurodegeneration.

Stress to the endoplasmic reticulum (ER) caused by disrupted Ca^2+^ homeostasis, glucose starvation, hypoxia, and oxidative stress including hyperhomocysteinemia [1,2,3] can lead to cell death [4,5,6]. ER stress also induces the accumulation of unfolded/misfolded proteins, which may elicit unfolded protein responses (UPR) through intracellular signaling pathways [7]. Previous studies have suggested that UPR are closely involved in the pathogenesis of AD and PD [8,9].

In this review, we examine the role of HMG-CoA reductase degradation protein 1 (HRD1), which is a ubiquitin E3 ligase, and that of the molecular chaperone protein-disulfide isomerase (PDI) in AD and PD. These proteins are all produced in the ER in response to stress. We discuss the therapeutic significance of HRD1, PDI, and chemical chaperones in treating neurodegenerative diseases.

## 2. ER Stress and UPR

**Figure 1:**
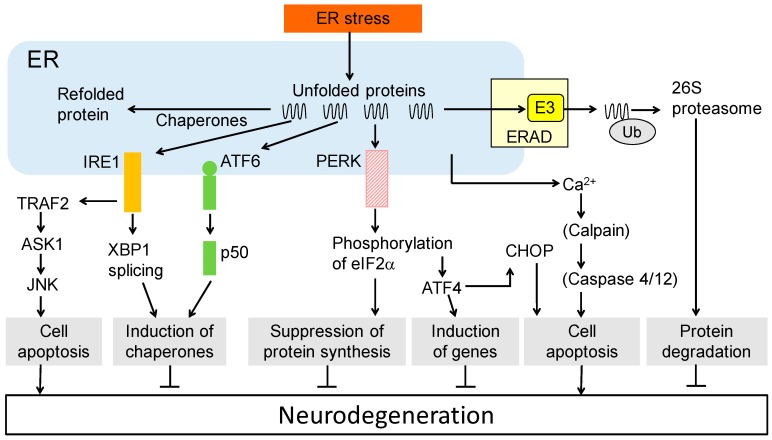
Unfolded protein responses and neurodegeneration. Unfolded protein responses related to endoplasmic reticulum (ER) stress and signaling pathways are shown. Each function positively and negatively affects the pathogenesis of neurodegeneration. ASK1: Apoptosis signal-regulating kinase 1; ATF: Activating transcription factor; ERAD: ER-associated degradation; CHOP: C/EBP-homologous protein; IRE1: Inositol-requiring enzymes 1; JNK: c-Jun N-terminal kinase; PERK: (PKR)-like ER kinase; TRAF2: TNF receptor-associated factor 2; XBP1: X-box binding protein 1;

The ER regulates intracellular Ca^2+^ concentrations, maturation of protein structures, and has crucial functions in the quality control of proteins. The accumulation of unfolded/misfolded proteins in the ER in response to stress induces the UPR, activating stress-related molecules such as inositol-requiring enzymes 1 (IRE1), activating transcription factor 6 (ATF6), and double-stranded RNA-activated protein kinase (PKR)-like ER kinase (PERK). Active IRE1 splices the X-box binding protein 1 (XBP1), which induces ER-resident chaperones (Figure 1).

A cleaved fragment of ATF6 (p50) and eIF2α phosphorylated by PERK also induces ER chaperones such as 78 kDa glucose-regulated protein (GRP78). Phosphorylated eIF2α also suppresses the biosynthesis of new proteins and the induction of cellular apoptosis (Figure 1) [4,10,11]. C/EBP-homologous protein (CHOP) genes are induced by phosphorylated eIF2α [12] and may be involved in apoptosis [13]. Furthermore, tribbles-related protein 3 (TRB3) was recently identified as a CHOP-inducible gene involved in cell death [14]. Additionally, the excessive/unfavorable accumulation of unfolded proteins leads to apoptotic cell death through the IRE1-TRAF2-ASK1-JNK pathway (Figure 1) [15]. ER-associated protein degradation (ERAD) is also involved in an UPR (Figure 1). The ER copes with aberrant proteins by ubiquitination and 26S proteasomal degradation. Cytoplasmic chaperones, such as heat shock proteins (HSP) HSP70 and HSP90, refold aberrant proteins. These ER functions may be important for cell survival. Several studies have suggested that disrupted activation of ER stress by a mutation in the presenillin gene downregulates the UPR and is involved in the pathogenesis of AD [16,17].

We previously identified two UPR-induced proteins: the E3 ubiquitin ligase HRD1 [18,19] and the molecular chaperone PDI [20]. In this review, we describe the cellular and molecular functions of these proteins. HRD1 and PDI play crucial roles in neuronal survival by acting as an E3 ubiquitin ligase and a molecular chaperone and may be involved in the pathogenesis of AD and PD (Figure 1). HRD1 and PDI may represent efficient tools in the pharmacological intervention of neurodegenerative diseases and are potential targets for novel AD and PD therapies.

## 3. HRD1: Identification and Characterization

We previously identified and cloned the human ER stress-responsive protein HRD1. In combination with the ubiquitin-activating enzyme E1 and the ubiquitin-associating enzyme E2, E3 catalyzes the ubiquitination and degradation of denatured/unfolded proteins. HRD1 binds, ubiquitinates, and degrades APP, reducing the formation of amyloid (Aβ) plaques [18,19,21].

Several UPR genes have been identified in the yeast *Saccharomyces cerevisiae* [22]. In an attempt to isolate and identify novel human UPR genes, we previously focused on the ERAD genes *Hrd1p*/*Der3p*, which exhibit E3 activity and are localized in the ER of yeast. Using bioinformatics, we identified human KIAA 1810 from the protein library that possessed an amino acid sequence with high homology to the Hrd1p protein in yeast. We cloned this protein and named and characterized it as described below [18,19].

Human HRD1 is ubiquitously expressed in the brain (CA1, substantia nigra, and Purkinje cells), lungs, liver, pancreas, kidneys, and skeletal muscle of mice. It is also expressed in the CA1 and CA4 regions of the human hippocampus and is not expressed in astroglial cells. HRD1 exhibits E3 ubiquitin ligase activity and exerts protective effects against ER stress-induced cell death [23].

Hypoxic stress promotes HRD1 expression in the cerebral cortex and striatum [24], implicating HRD1 in the response to and suppression of ER stress in the brain. Treating HEK293 cells with ER stress inducers (thapsigargin (a sarco/reticulum Ca^2+^-ATPase inhibitor) or tunicamycin (an *N*-glycosylation inhibitor)) markedly increases HRD1 expression [18,19]. *HRD1* mRNA expression in HEK293 cells are regulated by IRE1 pathways [25], which has also been demonstrated in yeast.

## 4. AD and HRD1

Two main hypotheses have been proposed for the pathology of AD: the Aβ hypothesis and the phosphorylated tau (P-tau) hypothesis. The Aβ hypothesis is based on the histological evidence of senile plaques and accumulation of Aβ, whereas the P-tau hypothesis is based on the appearance of neurofibrillary tangles and accumulation of the P-tau protein in the brain [26,27].

### 4.1. Aβ Hypothesis

Among several hypotheses on the pathogenesis of AD, the Aβ hypothesis has been well received [28] but is not yet generally accepted [29]. Aβ, composed mainly of Aβ_1–40_ and Aβ_1–42_, is generated from APP by the peptidase enzymes, β-secretase and γ-secretase [30,31,32,33,34]. Aβ induces the formation of oligomers, which leads to neuronal death [35,36,37,38]. To develop novel therapeutics for AD, extensive efforts have been made to identify molecules that can target and reduce the levels of Aβ, including γ-secretase inhibitors and vaccines against Aβ [39,40,41,42,43]. These efforts have not been successful, but the implantation of microglia/microglia-like cells into local areas of the brain may reduce Aβ levels _1–42_ in vivo [44,45]. Novel therapeutic targets or strategies are urgently needed.

### 4.2. HRD1: APP Ubiquitination and Reduction in the AD Brain

**Figure 2:**
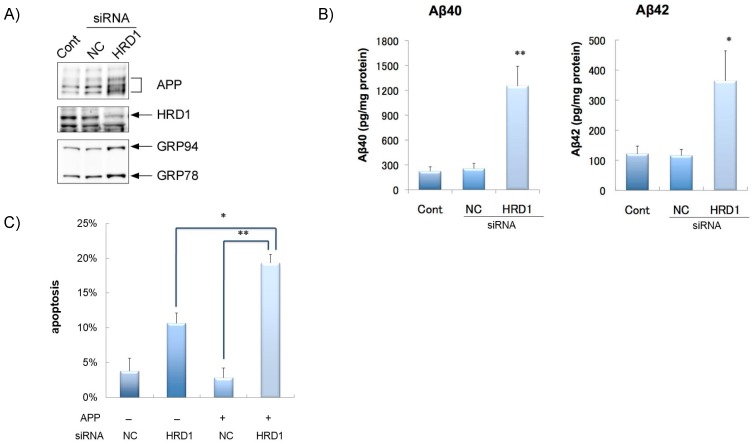
Amyloid precursor protein (APP) accumulation, amyloid plaques (Aβ) generation, and neuronal apoptosis by HMG-CoA reductase degradation protein 1 (HRD1) suppression in SH-SY5Y cells. (**A**) Induction of APP accumulation by HRD1 siRNA. SH-SY5Y cells stably expressing APP-FLAG were analyzed by western blotting with the indicated antibodies; (**B**) Aβ40 and Aβ42 were measured by sandwich ELISA using the culture medium from (**A**). Statistical analysis was performed with ANOVA. * *p* < 0.05; ** *p* < 0.01; Con: control, NC: non-target control, HRD1: treatment with siRNA-HRD1; (**C**) Cell apoptosis after treatment with HRD1 siRNA. SH-SY5Y cells stably expressing APP-FLAG were transiently transfected with NC or siRNA-HRD1. The cells were subjected to immunofluorescence staining with anti-cleaved caspase-3 antibodies. Staining was analyzed statistically. The percentage of apoptotic cells in three different areas was calculated. * *p* < 0.05; ** *p* < 0.01. NC: non-target control, HRD1: treatment with siRNA-HRD1.

We previously reported that HRD1 colocalizes with APP in mouse neurons, binds APP at proline-rich regions of HRD1, and ubiquitinates and degrades APP [21,46]. Overexpression of HRD1 reduces the generation of Aβ_1–40_ and Aβ_1–42_. In contrast, the suppression of HRD1 expression by small interfering RNA (siRNA) induces APP accumulation and neuronal death (Figure 2) [21].

In addition, the generation of Aβ_1–40_ and Aβ_1–42_ (Figure 2) is significantly enhanced. Thus, HRD1 ubiquitinates and degrades denaturated APP as well as unfolded proteins, suggesting that HRD1 affects APP-Aβ dynamics in the brains of AD patients.

It has not yet been established whether HRD1 functions normally in the AD brain. Solubilized HRD1 protein (by 1% NP-40 detergent) levels are lower in the postmortem cerebral cortex of AD patients than in the non-AD controls [21,46]. It was previously shown that HRD1 expression correlates negatively with Aβ_1–40_ and Aβ_1–42_ [47]. Although this study was limited regarding the number of specimens and consideration of the clinical states, these findings suggest that HRD1 participates in reducing Aβ levels, thereby suppressing the pathogenesis of AD. In contrast to the decreased soluble HRD1 protein levels in AD, *HRD1* mRNA expression has been shown to increase, suggesting a secondary compensation for the reduced protein levels. We aimed to clarify the mechanism of HRD1 protein insolubilization in the brain of AD patients; we examined whether Aβ, tau, ER stress, or oxidative stress, which are associated with AD pathology, induce HRD1 insolubilization. We found that oxidative stress causes insolubilization of HRD1 protein but not other AD-related stresses (Aβ, tau, and ER stress) [48]. Therefore, oxidative stress-induced HRD insolubilization may be involved in Aβ accumulation and in the pathogenesis of AD.

Further studies are required to determine whether HRD1 ubiquitinates and degrades phosphorylated tau proteins [49].

## 5. PD, HRD1, and Chaperones

PD is a common neurodegenerative disease with a movement disorder, particularly in the elderly. The pathological hallmark of PD is the loss of dopamine (DA)-containing neurons in the substantia nigra pars compacta (SNC), reducing DA levels in the striatum.

### 5.1. Role of ER Stress in the Pathogenesis of PD

Most cases of PD are sporadic, and an estimated 5%–10% of patients are monogenic. The genes involved in autosomal recessive PD are *Parkin* (*PARK2*), *PINK1* (*PARK6*), *DJ1* (*PARK7*), and *ATP13A2* (*PARK9*). The genes involved in autosomal dominant PD are *α-synuclein* (*PARK 1*/*4*), *LRRK2* (*PARK8*), and *UCHL-1* (*PARK5*) [50,51]. LRRK2 and α-synuclein are common risk factors for sporadic PD and have been identified as substrates for ERAD-related ubiquitin ligase E3. Furthermore, changes in ERAD-related E3, C-terminus of HSP70-interacting protein (CHIP), and Parkin levels have demonstrated that disturbances in the ERAD system are responsible for the onset of PD [52,53,54,55]. Thus, ER stress may be a causative factor for PD.

Parkin is an E3 ubiquitin ligase and ubiquitinates its own substrate, the misfolded Parkin-associated endothelin receptor-like receptor (Pael-R), suppressing cell death caused by the accumulation of Pael-R [56]. Parkin mutations reduce E3 activity, which leads to the accumulation of unfolded Pael-R and ER stress-induced cell death. Therefore, ER stress caused by the accumulation of Pael-R is relevant to the pathological mechanisms underlying autosomal recessive PD.

Previous studies have shown that Parkin-deficient mice exhibited no significant changes in dopaminergic neurodegeneration or the accumulation of Parkin substrates [57,58,59]. However, Parkin-knockout/Pael*-R* transgenic mice exhibited fewer dopaminergic neurons [60], suggesting that other E3s degrade accumulated Pael-R in the absence of Parkin as a compensatory mechanism. The co-operation of PINK1 and Parkin in the treatment of damaged mitochondrial degradation has already been reported [61,62]. Autophosphorylated PINK1 recruits Parkin to degrade damaged mitochondria. However, in PD, these events are averted by *PINK1* mutations. These findings implicate E3 Parkin in the onset of PD.

### 5.2. HRD1 and PD

We previously demonstrated that HRD1 is expressed in the SNC, particularly in DA-containing neurons [63]. Pael-R is also expressed in DA neurons of the SNC. We showed that HRD1 and Pael-R colocalize in the ER of dopaminergic SH-SY5Y cells. Pael-R binds to and is ubiquitinated by HRD1. The reduction of endogenous HRD1 by siRNA causes leads to the accumulation of Pael-R and activation of caspase-3. This demonstrates that Pael-R is also a substrate for HRD1 and Parkin [64]. We reported that Pael-R-induced cell death was suppressed by the overexpression of HRD1. α-synuclein is not a substrate of HRD1; therefore, HRD1 may be involved in the onset of PD through Pael-R but not through α-synuclein.

### 5.3. PDI, Chemical Chaperones, and PD

Molecular chaperones function in protein quality control, for example in the folding of misfolded/unfolded proteins that accumulate in the ER under stressful conditions such as oxidation and/or mutated gene products [65,66,67]. Molecular chaperones are essential for cell survival. Stress causes unfolded proteins to accumulate in the ER, which induces molecular chaperones such as Bip, calnexin, glucose-regulated protein 94 (GRP94), and PDI. We previously demonstrated the upregulation of PDI in response to hypoxia/brain ischemia and its protective effects against hypoxia/ischemia-induced apoptotic cell death [20]. These findings suggest that PDI suppresses the onset of Pael-R-related PD.

#### 5.3.1. PDI

PDI prevents thapsigargin- and tunicamycin-dependent neuronal apoptosis, suggesting that PDI suppresses ER stress-induced neuronal death [63,68]. PDI also inhibits MG132 (proteasomal inhibitor)-induced cell death, which indicates that protein ubiquitination and degradation participates in cell death [69]. A previous study reported that PDI suppresses Pael-R-induced neuronal death, suggesting that PDI functions as a chaperone to alleviate and rescue neuronal death in PD [69].

PDI catalyzes a thiol-disulphide exchange reaction that facilitates disulphide bond formation and a rearrangement reaction. In the PDI molecule, four cysteines form thioredoxin-like domains, which are involved in catalytic reactions. Although S-nitrosylation has been proposed as a physiological signal for neuronal nitric oxide [70], these cysteines are nitrosylated by NO donors, reducing the catalytic activity. We previously reported an increase in SNO-PDI levels in postmortem brains of AD and PD patients [69]. Yao et al. identified a relationship between nitrosative stress and sporadic PD [71]. Thus, the physiological and pathophysiological roles of cerebral nitric oxide have already been demonstrated. The S-nitrosylation of PDI may be involved in neuronal death, contributing to AD and PD pathogenesis [69].

#### 5.3.2. Chemical Chaperones

Chemical chaperones stabilize proteins and ameliorate the accumulation of unfolded proteins [72,73] to attenuate ER stress. Glycerol, dimethylsulfoxide (DMSO), and 4-phenyl-butyrate (4-PBA) have been identified as chemical chaperones. 4-PBA has been employed in clinical trials for sickle-cell anemia [74], β-thalassemia [75], and cystic fibrosis [76]. We previously demonstrated that 4-PBA promoted the correct folding of unfolded Pael-R and suppressed cell death caused by Pael-R accumulation [77,78]. A synthetic derivative of 4-PBA, 4-(4-methoxyphenyl)butanoic acid was better at inhibiting protein aggregation and ER stress-induced neuronal death than 4-PBA [79]. Tauroursodeoxycholic acid (TUDCA) is a chemical chaperone that inhibits Aβ-induced cell death [80,81,82]. However, the therapeutic effects of chemical chaperones for AD and PD have only been investigated in vitro so far.

## 6. Potential Therapeutic Strategies for AD and PD

**Figure 3:**
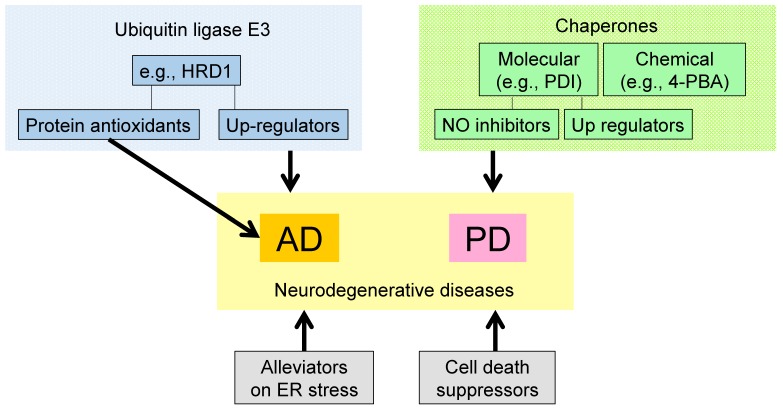
Strategies for developing therapeutics for neurodegenerative diseases. Protein antioxidants and upregulators of ubiquitin ligase E3 (e.g., HRD1) could be therapeutic targets for AD. Upregulators of molecular chaperones, inhibitors of nitrosylation of PDI, and chemical chaperones could be therapeutic targets for PD. In addition, alleviators of ER stress and suppressors of cell death could be therapeutic targets for neurodegenerative diseases. AD: Alzheimer’s disease, PD: Parkinson’s disease.

In this review, we suggest that alleviators of ER stress and suppressors of ER stress-induced neuronal cell death represent potential therapeutic targets for AD and PD (Figure 3).

HRD1 and molecular chaperones against AD and PD are possible candidates for the discovery of therapeutics against neurodegenerative diseases. Induction of molecular chaperones would be one of the useful strategies for ameliorating the diseases. Interestingly, BiP inducer X (BIX) was shown to protect against neuronal cell death caused by ER stress by inducing the molecular chaperone, GRP78 [83]. Furthermore, chemical chaperones, which can reduce aggregated protein accumulation may be also beneficial for the treatments. However, considering the usage of such a kind of drugs, there currently have no evidence for the clinical use without side effects. Therefore, future analysis may be required for elucidating such compounds. Moreover, protein antioxidants and NO inhibitors may also be efficient against AD and PD. Autophagy, another protein degradation pathway in addition to ubiquitination, has potential as a target for novel AD and PD therapies [84,85,86].

Molecules that decrease and/or deplete Aβ and α-synuclein are currently being investigated. A vaccine against Aβ has not yet been successfully developed, but an injection of microglial/microglia-like cells into the brain appears to remove Aβ [44,45]. Peptide synthesis of pathogenic Aβ_1–42_ oligomer aggregate inhibitors may be successful [87]. It is important to identify patients with mild cognitive impairment during early stages of the disease, and this requires probes for neuroimaging and suitable clinical biomarkers. Further efforts are needed to develop both therapeutics and prophylactics.

We are hopeful that a local injection of iPS cells with the potential to differentiate into acetylcholine- or DA-containing neurons into pathological regions of the hippocampus or substantia nigra will successfully treat the symptoms of neurodegenerative diseases in the future.

## 7. Conclusions

ER stress/UPR may be involved in the pathogenesis of AD and PD. Therefore, HRD1 and PDI represent promising targets for the development of novel AD and PD therapies. In addition, chemical chaperones are potential therapeutic candidates for these diseases. Currently, development of drugs targeting unfolded proteins are still in the nascent stage. Furthermore, there are no clinically useful drugs that can modify activity of HRD1 and PDI. We believe that development of these drugs will be a useful strategy for future treatment of AD and PD.
